# Structural and activity characterization of human PHPT1 after oxidative modification

**DOI:** 10.1038/srep23658

**Published:** 2016-04-01

**Authors:** Daniel R. Martin, Priyanka Dutta, Shikha Mahajan, Sameer Varma, Stanley M. Stevens

**Affiliations:** 1 Department of Cell Biology, Microbiology and Molecular Biology, University of South Florida, Tampa, FL 33620, USA

## Abstract

Phosphohistidine phosphatase 1 (PHPT1), the only known phosphohistidine phosphatase in mammals, regulates phosphohistidine levels of several proteins including those involved in signaling, lipid metabolism, and potassium ion transport. While the high-resolution structure of human PHPT1 (hPHPT1) is available and residues important for substrate binding and catalytic activity have been reported, little is known about post-translational modifications that modulate hPHPT1 activity. Here we characterize the structural and functional impact of hPHPT1 oxidation upon exposure to a reactive oxygen species, hydrogen peroxide (H_2_O_2_). Specifically, liquid chromatography-tandem mass spectrometry was used to quantify site-specific oxidation of redox-sensitive residues of hPHPT1. Results from this study revealed that H_2_O_2_ exposure induces selective oxidation of hPHPT1 at Met95, a residue within the substrate binding region. Explicit solvent molecular dynamics simulations, however, predict only a minor effect of Met95 oxidation in the structure and dynamics of the apo-state of the hPHPT1 catalytic site, suggesting that if Met95 oxidation alters hPHPT1 activity, then it will do so by altering the stability of an intermediate state. Employing a novel mass spectrometry-based assay, we determined that H_2_O_2_–induced oxidation does not impact hPHPT1 function negatively; a result contrary to the common conception that protein oxidation is typically a loss-of-function modification.

Phosphorylation of serine, threonine and tyrosine residues is vital to various cellular processes including signal transduction and cell metabolism; however, the role of histidine phosphorylation, specifically in the context of mammalian cell signaling, is relatively unexplored. Over the past several decades, several protein targets of histidine phosphorylation in eukaryotic systems have been identified including ATP-citrate lyase^1^, G protein (beta subunit)^2^, histone H4^3^, the potassium ion channel KCa3.1^4^ as well as the phosphorylated enzyme intermediates involved in phosphoryl group transfers to metabolites^5^,^6^,^7^. The addition or removal of the histidine phosphoryl group in these proteins is mediated through the action of histidine kinases or phosphatases, respectively, where only a few eukaryotic enzymes have been identified to date. These enzymes include nucleoside diphosphate kinase^8^ as well as phosphohistidine phosphatase 1 (PHPT1)^9^.

PHPT1 was identified in porcine liver in 2002 as a 14 kDa protein that possesses phosphatase activity for phosphohistidine residues^9^ and has also been shown to catalyze the dephosphorylation of phosphoramidate^10^ as well as phospholysine^11^. Since its discovery, PHPT1 has been shown to be involved in regulating the levels of known mammalian phosphohistidine targets. For example, PHPT1 plays a negative regulatory role when removing the histidine phosphoryl group in the potassium ion channel, KCa3.1^4^. Additionally, PHPT1 overexpression in neuroblastoma and primary neurons decreases ATP-citrate lyase activity and cell viability^12^. PHPT1 was also demonstrated to participate in G-protein mediated cell signaling in islet β cells^13^. PHPT1 is part of the Janus family of proteins, and the only phosphohistidine phosphatase identified within this family in humans to date according to family domain classification available on the Universal Protein Resource (www.uniprot.org). Janus family proteins are best characterized in *Drosophila melanogaster* where they are involved in sex differentiation; however, their role as phosphohistidine phosphatases is still unclear^14^.

The human PHPT1 (hPHPT1) structure is composed of six β-strands flanked by two α-helices^15^. The substrate-binding site is positioned between two loops, one between α1 and β4, and the other between β5 and α2^15^. This site is composed of multiple residues that have been shown to be important for substrate binding, including Glu51, Tyr52, His53, Tyr93, and Met95^15^. Additionally, the NH groups of Ala54, Ala96, and His53 and the OH group of Ser94 are involved in stabilizing phosphate binding to the active site of hPHPT1^16^.

While the residues important for hPHPT1 activity and substrate binding have been characterized, there have been no studies concerning the structural and functional outcomes of post-translational modification (PTM) or chemical modification to this protein. To the best of our knowledge, N-terminal protein acetylation is the only PTM identified on hPHPT1^17^. Here, we investigate the impact of oxidation, specifically through the known reactive oxygen species (ROS), hydrogen peroxide (H_2_O_2_), on hPHPT1 structure and related activity using mass spectrometry and computational methods. ROS are highly reactive molecules that induce functional alterations in the cell and their overproduction is linked to fundamental biological processes, such as aging as well as pathophysiological processes of disease^18^. Using mass spectrometry, H_2_O_2_-induced global oxidation of recombinant hPHPT1 as well as site-specific quantitation of protein oxidation was performed. Additionally, a mass spectrometry-based activity assay was developed to determine the functional impact of hPHPT1 oxidation and to provide additional specificity compared to results from a colorimetric activity assay that employed a non-specific phosphatase substrate. Moreover, computational studies using molecular dynamics simulations were carried out to investigate structural outcomes from selective methionine oxidation of hPHPT1. The results from this combined experimental and computational study provide new insight into the significance of hPHPT1 as a target of ROS and highlight the importance of elucidating the correlation of structural to functional diversity brought about by protein modifications.

## Materials and Methods

### hPHPT1 oxidation

Human recombinant PHPT1 (referred to as hPHPT1) was obtained from Sino Biological Inc. (catalog number: 12473-H07E) and stored at a concentration of 1 mg/ml in phosphate buffered saline (PBS). This stock solution was used to create a 20 μM solution diluted in PBS for subsequent oxidation characterization and activity analysis using mass spectrometry. From this solution, 46 μl were combined with either 46 μL of water or H_2_O_2_, at various concentrations, to create a 10 μM solution of hPHPT1 with H_2_O_2_. The solution was then incubated in a heat bath at 37 °C for two hours to allow oxidation to occur. Each sample was then processed for either the phosphohistidine phosphatase activity assay using mass spectrometry or para-nitrophenyl phosphate assay described below. Two μg of protein, taken directly from the same samples used for the phosphohistidine phosphatase activity assay, were used for oxidative site mapping by mass spectrometry.

### Oxidation site mapping using mass spectrometry

The hPHPT1 samples for oxidation site mapping by mass spectrometry were first reduced and alkylated with DTT and iodoacetamide, respectively, followed by dilution in a digestion buffer containing 25 mM ammonium bicarbonate. Protein digestion with trypsin (Promega) was carried out overnight at 37 degrees C and then the samples were desalted using Hypersep C18 columns (Thermo Scientific) columns. Following centrifugation of the samples under vacuum until dryness, samples were resuspended in 0.1% formic acid in water for mass spectrometric analyses. Peptides generated from the trypsin digestion were separated on an Acclaim PepMap C18 (75 μm × 50 cm) UPLC column (Thermo) using an EASY-nLC 1000 with a gradient time of 60 min (2–40% acetonitrile in 0.1% formic acid). Mass spectrometric analysis was performed by a hybrid quadrupole-Orbitrap instrument (Q Exactive Plus, Thermo), using a top 10 data-dependent acquisition method with a dynamic exclusion time of 20 seconds. Full scan and MS/MS resolution was 70,000 and 17,500, respectively.

High-resolution mass spectrometric data were searched against the Uniprot human database using the MaxQuant (version 1.5.0.3, maxquant.org) search algorithm. Variable modifications included mono-oxidation of methionine, cysteine, and tyrosine, di-oxidation of methionine, tryptophan, and cysteine, tri-oxidation of cysteine, and carbamidomethylation of cysteine. MaxQuant search parameters included a first search peptide tolerance of 20 ppm and a main search peptide tolerance of 4.5 ppm. Global-scale automated modification quantitation was performed using the MaxQuant calculation of mod/base ratio for each oxidative modification and then normalized to the most oxidized amino acid residue. Identifications were accepted at a protein and peptide false discovery rate of less than 1% and overall localization probabilities of ≥95% for modified peptides. The MaxQuant viewer and Scaffold (version 4.4.3, Proteome Software) were used to visualize annotated MS/MS spectra to confirm modification-specific locations. Xcalibur Qual Browser (ThermoScientific; version 2.2) was then employed to further quantify residue-specific oxidation using high mass accuracy, extracted ion chromatogram-based (XIC) quantitation for residues exhibiting significant changes in MaxQuant mod/base ratio values. In this approach, area-under-the-curve (AUC) values were determined for both the oxidized and non-oxidized peptide ions based on the m/z values of the monoisotopic and A + 1 peak using a mass tolerance of 5 ppm and a precision of 5 decimal places for chromatogram reconstruction. Ratios of the oxidized: non-oxidized peptides were determined for each replicate and then averaged for each treatment. These average ratios were then used to determine fold changes relative to the amount of oxidation that occurred in the control groups. Statistical significance was established at p < 0.05 using the Student’s t-test (two-tailed, homoscedastic) with at least three replicates for all experiments.

### Molecular Dynamics

The MD simulations of hPHPT1 and its oxidized form were carried out under identical conditions. The initial coordinates of hPHPT1 were taken from the NMR structure of its apo-state (PDB ID: 2OZX)^16^, and those of its oxidized form were constructed by substituting Met95 in the NMR structure by methionine sulfoxide. Both forms of hPHPT1 were simulated in cubic boxes containing 13,130 water molecules and discrete K^+^ and Cl^−^ ions, with their specific numbers corresponding to ionic strengths of 100 mM. The ionization states of the titratable groups in hPHPT1 were assigned according to the NMR data, and the charge neutrality of the unit cells was set by choosing appropriate differences between the numbers of K^+^ and Cl^−^ ions. Both forms of hPHPT1 were simulated under isobaric-isothermal boundary conditions. Pressure was maintained at 1 bar using extended ensemble approach^19^ with a coupling constant of 1 ps and a compressibility of 4.5 × 10^−5^ bar^−1^. Temperature was maintained at 310 K using the velocity rescale approach^20^ with a coupling constant of 1 ps. Electrostatic interactions were computed using the particle mesh Ewald scheme^21^ with a Fourier grid spacing of 0.15 nm, a sixth-order interpolation, and a direct space cutoff of 10 Å. Van der Waals interactions were computed explicitly for inter atomic distance up to 10 Å. The bonds in proteins were constrained using the P-LINCS algorithm^22^, and the geometries of the water molecules were constrained using SETTLE^23^. These constraints permitted use of an integration time step of 2 fs. The protein and ions were described using OPLS-AA parameters^24^, and the water molecules were described using TIP4P parameters^25^. We used GROMACS version 4.5.3 for all MD simulations^26^.

### Phosphohistidine synthesis

To synthesize the phosphohistidine-containing peptide that is a reported substrate of PHPT1, phosphoramidate (5.4 mg) was first combined with the peptide (4.6 mg), suc-AHPF-pNA, (MW 690.7 Da, BaChem) in 200 μL of H_2_O at pH 9 with NaOH. This solution was then shaken for 5 hours at room temperature to allow the phosphorylation reaction to occur. Immediately following the reaction, liquid chromatography was performed on the phosphohistidine-containing peptide. This was achieved using a glass pipette filled with Dowex 3 anion exchange resin, stopped with 2 mm of cotton. The resin was washed three times with 200 μl of 25 mM Tris buffer pH 9 to equilibrate the column prior to sample loading. A pipette stopper was used to speed up the chromatography movement and make sure the wash had completely flowed through before any sample was introduced. The full volume of approximately 205 μl of the phosphohistidine sample was then loaded onto the column followed by sample saturation on the column through positive pressure induced by the pipette stopper. Sample was collected and stored at 4 °C prior to mass spectrometry analyses with longer storage times resulting in greater histidine phosphorylation. This procedure yielded 35–40% histidine phosphorylation as approximated by mass spectrometric intensities of the phosphorylated and non-phosphorylated peptide ions in the product mixture.

### Mass spectrometric phosphohistidine phosphatase activity assay

The hPHPT1 samples treated with H_2_O_2_ treatments or H_2_O and PBS as a control were added to a custom reaction buffer (5:95% v/v acetonitrile: H_2_O and 20 mM ammonium bicarbonate, pH 8.5, referred to as RB) to reach a final concentration of 0.37 μM and the phosphohistidine peptide solution was added to reach a final concentration of 2.4 mM. The solution was then vortexed for 5 s and analyzed by direct infusion combined with ESI-MS, using a hybrid linear ion trap-Orbitrap (Orbitrap XL, ThermoFisher) mass spectrometer to determine mass spectrometric peak intensities of the phosphorylated and non-phosphorylated peptide for 30 min in RB. The instrument was tuned to the phosphorylated peptide peak at m/z 771.2. The following electrospray ionization (ESI) source settings allowed for optimal signal of the phosphohistidine-containing peptide: Source Voltage: 5.00 V, Sheath Gas Flow Rate: 8 (arb), Capillary Voltage: 46.00 V, Capillary Temp: 275 °C, Tube Lens Voltage: 135 V. Samples were infused directly using a flow rate of 3 μl/min. These optimized settings were used for all experiments utilizing the phosphohistidine peptide on this instrument in order to minimize API source-mediated phosphate loss from the phosphopeptide substrate^27^. Non-phosphorylated and phosphorylated peptide sequences were confirmed used collision-induced dissociation with an isolation width of 2.0, a normalized collision energy of 33, an activation Q of 0.25, and an activation time of 30 ms in the linear ion trap followed by high resolution detection of the fragment ions in the Orbitrap mass analyzer. Full scan MS and MS/MS spectra were acquired in centroid mode.

After a delay of approximately 1 min between the start of the reaction and the direct infusion of the sample, intensity measurement was performed on several peaks corresponding to the non-phosphorylated peptide including m/z 691, 713, and 729 ([M + H]^+^, [M + Na]^+^, and [M + K]^+^, respectively) in comparison with m/z 771, 793, and 809 peaks representing the phosphorylated peptide ([M_phospho_ + H]^+^, [M_phospho_ + Na]^+^ and [M_phospho_ + K]^+^, respectively) over the time course of 30 min. Full MS spectra were averaged over 5 min time intervals (0–5 min, 5–10 min, etc.) and the intensities of each peak relating to the phosphorylated and non-phosphorylated peptide were then used to calculate the ratio of total phosphorylated: total non-phosphorylated peptide (Intensities of all the phosphorylated peptide peaks/intensities of all non-phosphorylated peptide peaks). The negative fold change, which corresponds to the conversion of the phosphorylated peptide by hPHPT1, was calculated and compared to the average ratio of the non-treated phosphohistidine peptide over all time points to account for any phosphohistidine loss during injection.

### Colorimetric phosphatase activity assay

The hPHPT1 samples were treated and prepared similarly to the previous assay (see Methods: h*PHPT1 oxidation*). However, aliquots of 10 μg of total hPHPT1 were used for each treatment. The reaction buffer was prepared according to Gong *et al*. using a total volume of 300 μl, per sample, containing 50 mM Tris/HCl and 5 mM DTT where the pH was increased to 8.0 with NaOH^16^. The concentration of para-nitrophenyl phosphate (*p*NPP; MP Bio) used was 5 mM and was added to the reaction buffer immediately before being plated. The final concentration of hPHPT1 in the solution volume (300 μl) was 2.1 μM (10 μg) for each sample. All samples were kept one ice until plated in a 96 well plate. The reaction buffer and hPHPT1 samples were added to the well in triplicates where 100 μl were plated per replicate. The plate was then immediately added to the plate reader (heated to 37 ^o^C) to begin the reaction after the initial absorbance measurement. The absorbance was measured at 405 nm for the non-phosphorylated product, *p*-nitrophenolate, and this measurement was taken every 3 min over the course of 2 h. Concentrations were determined using the molar extinction coefficient^16^ of 17.8 mM^−1^cm^−1^ and the light path length calculated based on the average radius of the wells (r = 4 mm) and quantity of reaction mixture (100 μl) in the well.

Enzyme kinetic analysis was performed for the non-oxidized (non-treated) hPHPT1 samples and the 500 μM H_2_O_2_-treated hPHPT1 samples. A substrate concentration range of 0.8 mM – 40 mM pNPP was used to obtain initial reaction velocities for Michaelis-Menten kinetics. Initial reaction velocities were calculated using the slope of the most linear portion of the curve created by plotting the amount of non-phosphorylated substrate generated over time. Initial reaction velocities and pNPP concentrations were then analyzed using Prism, Graph Pad (version 6.07) by fitting to the Michaelis-Menten equation. Average K_m_ and V_max_ values for each non-oxidized and oxidized hPHPT1 were reported with a 95% confidence interval. The k_cat_ of each sample was then calculated using V_max_ values and the final concentration of hPHPT1 used in the assay.

## Results

### H_2_O_2_ induced oxidation of hPHPT1

**Figure 1:**
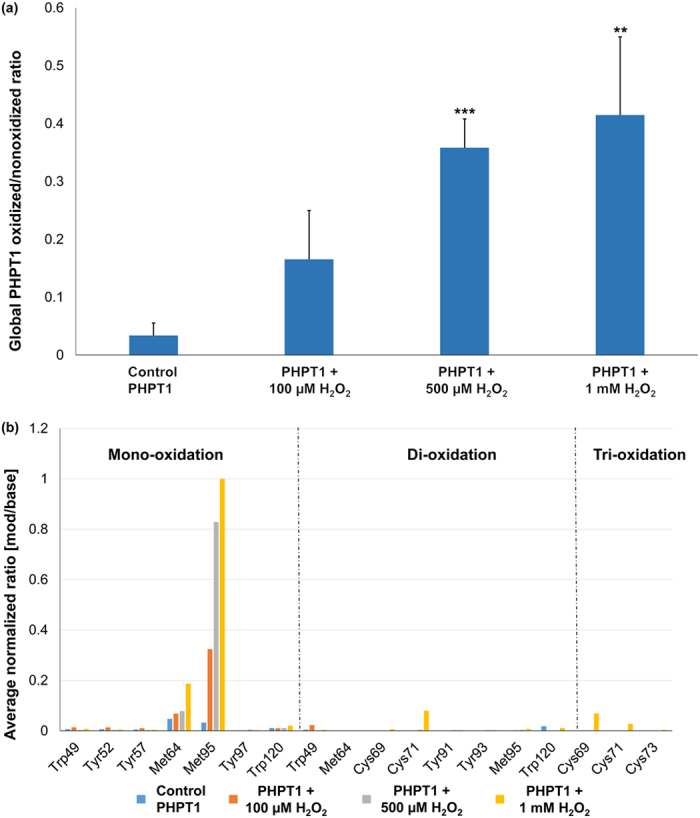
Global and amino acid-specific oxidation of hPHPT1. (**a**) Global hPHPT1 oxidized/non-oxidized ratios following treatment with H_2_O_2_ at various concentrations were determined using the sum of intensities for all oxidized peptides/sum of intensities for non-oxidized peptides based on identified hPHPT1-derived peptides from MaxQuant. Error bars represent standard deviation. **indicates p < 0.01, ***indicates p < 0.001 using Student’s t-test comparison of treatment to control group (**b**) Control and H_2_O_2_-treated hPHPT1 were digested with trypsin and analyzed by LC-MS/MS. Mass spectrometric data were analyzed by MaxQuant and average mod/base values for oxidized peptides identified were normalized to the largest value (corresponding to mono-oxidation of Met95) and displayed for relative comparison of amino acid residues susceptible to oxidation (Trp, Tyr, Met, and Cys). Residues shown are present on hPHPT1-derived tryptic peptides from MaxQuant filtered at 1% FDR and >95% overall localization probability.

**Figure 2:**
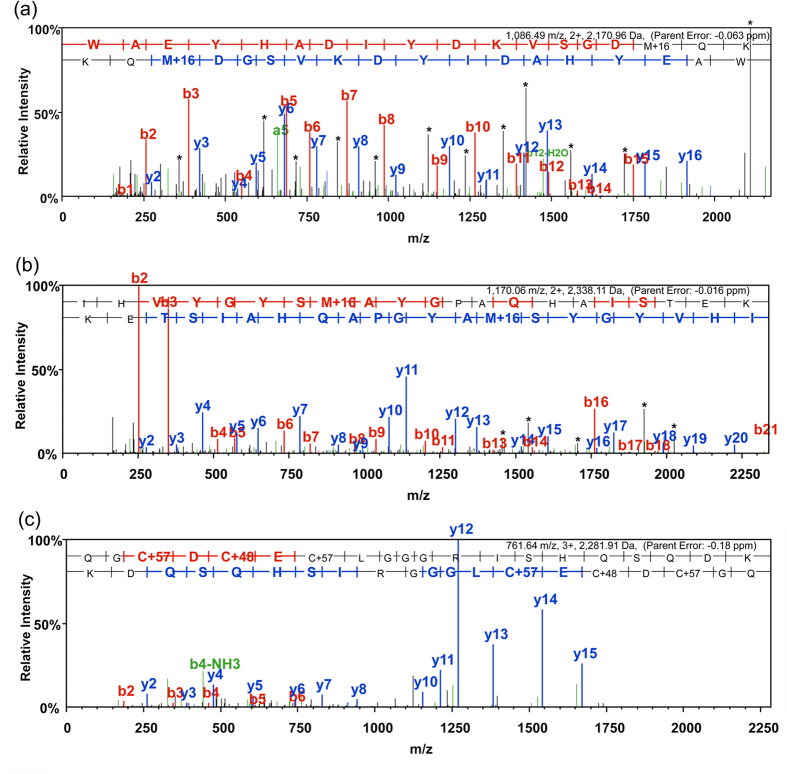
Representative MS/MS spectra of tryptic peptides derived from oxidized hPHPT1 used to identify the sequence and localize the oxidation site of (a) Met64 mono-oxidation, (b) Met95 mono-oxidation, (c) Cys71 tri-oxidation. Annotated MS/MS spectra were exported from Scaffold where b- and y-type ions represent location of amide bond cleavage from CID as shown in the sequence above the MS/MS spectrum. Peaks in black and marked with * of mono-oxidized methionine-containing peptides correspond to a neutral loss of 64 Da (-CH_4_SO) from the fragment ions.

The specific location and extent of protein oxidation induced by H_2_O_2_ was determined by LC- tandem mass spectrometry (MS/MS). Recombinant hPHPT1 was incubated for two hours in a heat bath at 37 °C with 0 μM, 100 μM, 500 μM, and 1 mM H_2_O_2_ (in triplicate). After LC-MS/MS analysis of trypsin-digested hPHPT1 from each treatment group, the mass spectrometric data were searched against the Uniprot human database with mono- and di-oxidation of Met, Cys, Tyr, and Trp as well as tri-oxidation of Cys set as variable modifications. hPHPT1 sequence coverage ranged from 95% to 99% and included all Met, Cys, Tyr, and Trp residues that were analyzed for oxidation quantitation. Using the MaxQuant intensity values for hPHPT1-derived peptides, the ratio of total oxidized peptide intensity/nonoxidized peptide intensity was calculated for each treatment group. As shown in Fig. 1a, a H_2_O_2_ concentration-dependent increase in hPHPT1 oxidation was observed. Furthermore, we used the MaxQuant value, mod/base, to assess the amount of residue-specific oxidation of Met, Cys, Tyr, and Trp residues. This analysis showed that Met95 was the most prominent site of H_2_O_2_ –induced oxidation, followed by Met64 (Fig. 1b). Additionally, di- and tri-oxidation of Cys71 was detected but only in the 1 mM H_2_O_2_ treatment group and at lower mod/base values than the methionine residues. Annotated MS/MS spectra (exported from Scaffold) for both Met64- and Met95-containing tryptic peptides as well as tri-oxidation of the Cys71-containing peptide are shown in Fig. 2. The MS/MS spectra allowed for confirmation of peptide sequence and oxidation site localization as well as peptide retention times used for manual extracted ion chromatogram (XIC)-based quantitation of oxidation with high mass accuracy.

**Figure 3:**
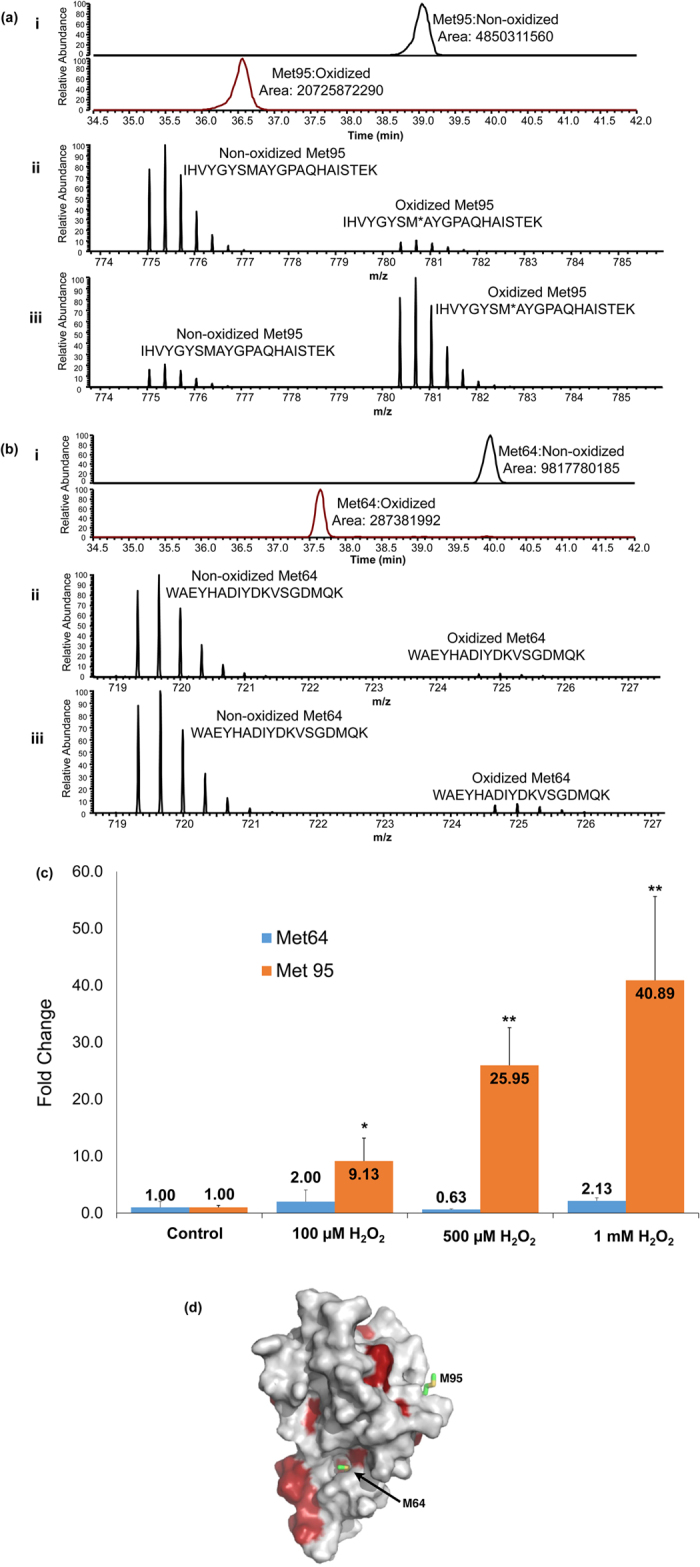
Quantitation of methionine oxidation in H_2_O_2-_treated hPHPT1. Representative accurate mass-based (<5 ppm) extracted ion chromatograms (XIC) corresponding to 1 mM H_2_O_2_-treated samples were generated for the Met95- (**a**)(i) and Met64-containing (**b**)(i) tryptic peptides. Superimposed full scan mass spectrum reflecting the relative abundance of the non-oxidized peptide compared to the oxidized cognate peptide in control hPHPT1 (untreated) for the Met95- (**a**)(ii) and Met64-containing (**b**)(ii) peptides. Overlay (3D) was performed in the Qual Browser (Thermo) data viewer followed by spectrum normalization to the largest peak in the scan with multiple scans normalized all the same. Spectrum reflecting the same peptides following 1 mM H_2_O_2_ treatment for the Met95- (**a**)(iii) and Met64-containing (**b**)(iii) peptides. (**c**) Fold change increase in Met95 and Met64 oxidation following treatment with H_2_O_2_ at various concentrations. The average AUC across replicates was used to determine fold change of oxidation for that treatment. Error bars represent standard deviation. *Indicates p < 0.05; **Indicates p < 0.01 using the Student’s t-test (**d**) NMR structure of hPHPT1 depicting the Met95 and Met64 residue location. Met64 is buried in a cavity that is partly hydrophobic (red indicates hydrophobic residues) while Met95 is surface-exposed.

Given the methionine residues of hPHPT1 were identified as a H_2_O_2_-induced oxidation target based on our unbiased screening approach using MaxQuant, XIC-based quantitation of oxidation at each of the two Met residues, Met64 and Met95, was performed with high mass accuracy in order to confirm and quantify oxidation with higher specificity. Representative XICs as well as the full scan mass spectrum averaged over the chromatographic peak width are shown for the Met95- and Met64-containing tryptic peptide in Fig. 3a,b, respectively. Area-under-the-curve (AUC) values from high mass accuracy-based XICs were then used to determine the relative abundance of oxidized peptides compared to non-oxidized peptides. The Met95-containing tryptic peptide showed a significant increase in oxidation between the control and all H_2_O_2_ treatments (Fig. 3c). An approximate 9-, 26-, and 41-fold increase in oxidation of Met95 was observed after 100 μM, 500 μM, and 1 mM H_2_O_2_ treatment, respectively. The Met64–containing tryptic peptide revealed lower levels of oxidation (up to 2-fold increase) and none significantly different from untreated hPHPT1 (Fig. 3c). The Met64-containing peptide was detected predominantly with one missed cleavage by trypsin; however, the peptide containing Met64 with no missed cleavage was also used for XIC-based quantitation. We note that the relatively higher oxidation of Met95 compared to that of Met64 is also consistent with the 3D structure of hPHPT1. As shown in Fig. 3d, while Met95 is exposed to solvent, Met64 is mostly buried and flanked by hydrophobic residues.

The significance of selective Met95 oxidation stems from the observation that it is located at hPHPT1’s substrate-binding site and that it has been shown to be important to substrate binding^28^. It is, therefore, plausible that oxidation of Met95 could modulate substrate binding and/or phosphatase activity.

### Effect of Met95 oxidation on the structure and dynamics of hPHPT1

All atom molecular dynamics (MD) simulations were employed to predict the response of Met95 oxidation on the structure and dynamics of hPHPT1. The study was restricted to hPHPT1’s apo-state as currently there isn’t sufficient experimental data to model the substrate-bound state. The basic strategy is to subject both oxidized and non-oxidized forms of hPHPT1 to MD simulations in explicit solvent and then systematically analyze the difference between them. Instead of generating one single MD trajectory, three separate 100 ns long MD trajectories were generated for each of the two forms – the motivation behind this was to enhance search in conformational space. We then combined the final 50 ns of the three trajectories of each form to obtain representative conformational ensembles for the two forms, 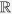 and 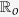.

These ensembles were then compared against each other to obtain the response of Met95 oxidation on the structure/dynamics of hPHPT1’s apo state. Instead of comparing summary statistics of these ensembles against each other^29^,^30^, we compared them directly against each other and obtained a quantitative estimate for the shift in conformational density, 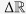. A direct comparison is preferred over a comparison of summary statistics as it circumvents the issue concerning the selection of representative conformations from rugged potential energy surfaces. A further advantage of comparing ensembles directly is that the resulting quantification naturally embodies differences in conformational fluctuations.

Quantitative estimates were obtained for the oxidation-induced shifts in conformational density, 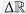, using a method we developed recently^31^,^32^,^33^. For a given pair of ensembles, this method returns a quantitative estimate of 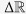 that we refer to as discriminability, 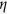. This quantity is normalized and bounded, that is, 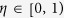, and it takes up a value closer to unity as the difference between the ensembles increases. We determined *η* separately for each of the 125 residues in the hPHPT1 between their representative ensembles in the oxidized and non-oxidized forms. Each ensemble was represented by 3001 conformations. These conformations were extracted at regular intervals of 50 ps from the combined 150 ns trajectory of each form. Note that all of the selected conformations are least square fitted on to the NMR structure. Structure fitting was necessary to remove the bias of *η* against whole molecule rotation and translation, as that was not the goal of this comparison.

**Figure 4:**
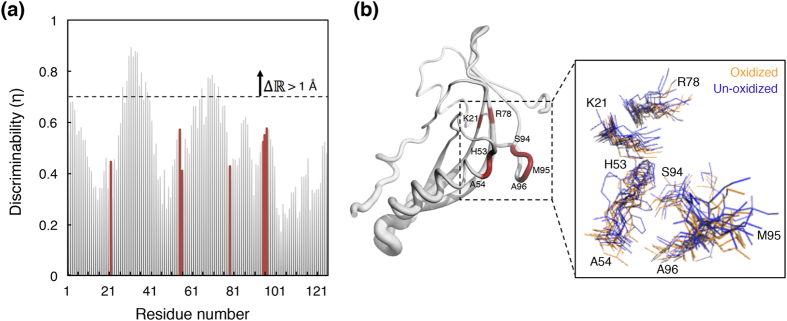
Effect of Met95 oxidation on the conformational density of hPHPT1. (**a**) The oxidation-induced conformational density shifts, 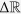, are shown individually for each of the 125 amino acids of hPHPT1, and indicated by a normalized quantity 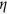 (discriminability) that takes up values closer to unity for progressively larger shifts. The residues that have been implicated to contribute to the catalytic activity of hPHPT1 are highlighted in red. The dashed horizontal line denotes the 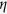 value beyond which the 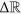 is equivalent to a center-of-mass (CoM) deviation of 1 Å. (**b**) NMR structure of hPHPT1 depicting 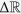 in terms of the thickness of the backbone trace. The residues highlighted in red are the same residues highlighted in red in (**a**). The inset provides a perspective of the conformational densities of these selected residues through the use of twelve conformations selected randomly from the oxidized and non-oxidized forms.

Figure 4 shows the oxidation-induced shifts in the conformational density of hPHPT1. It was found that for most residues *η* < 0.69, which, in Euclidian space is equivalent to a center of mass (CoM) deviation smaller than 1 Å. The only contiguous sequence of residues whose *η* > 0.69 was that of loop L2 (residues 29–39), which is distant from the catalytic site. The relatively larger *η* values for the residues in this loop is still equivalent to centers of mass deviations <2 Å. The set of residues in the catalytic site, Lys21, His53, Ala54, Arg78, Ser94, Ala96, that are known experimentally to contribute to hPHPT1’s activity^15^,^16^, underwent only negligible (<0.8 Å) oxidation-induced changes in conformational density. The backbone amino groups of His53, Ala54, and Ala96 as well as the side chains of both His53 and Ser94 remain oriented toward the catalytic site and explored conformational spaces similar to those in the non-oxidized form. Residues Lys21 and Arg78 that have been implicated in substrate anchoring^15^, and/or in stabilizing transition states^16^, retain the conformational space they explore in the non-oxidized form. The structure/dynamics of Met95 also remains unaffected by its oxidation.

In all, these MD simulations predicted that Met95 oxidation induces only a minor change in the structure/dynamics of the apo-state of hPHPT1. Therefore, if Met95 oxidation affects hPHPT1 activity, then it would do so by altering the stability of the ligand-bound transition state.

### Effect of H_2_O_2_ induced oxidation on hPHPT1 activity

To determine the effect of H_2_O_2_-induced oxidation on hPHPT1 activity, we utilized a novel mass spectrometry-based assay. In this assay, we measured the amount of a phosphohistidine-containing peptide substrate that remained after a specified time period of hPHPT1 treatment for both the non-oxidized and oxidized forms of the enzyme. Non-oxidized hPHPT1 should remove the phosphoryl group from phosphohistidine whereas oxidation, presumably through selective oxidation of Met95 that is within the substrate binding region, could induce changes in hPHPT1 phosphatase activity. The mass spectrometry assay allowed for detection and relative quantitation of both phosphorylated and non-phosphorylated forms of the peptide substrate over a defined period of time in order to identify significant changes in hPHPT1 function that could occur due to oxidation.

**Figure 5:**
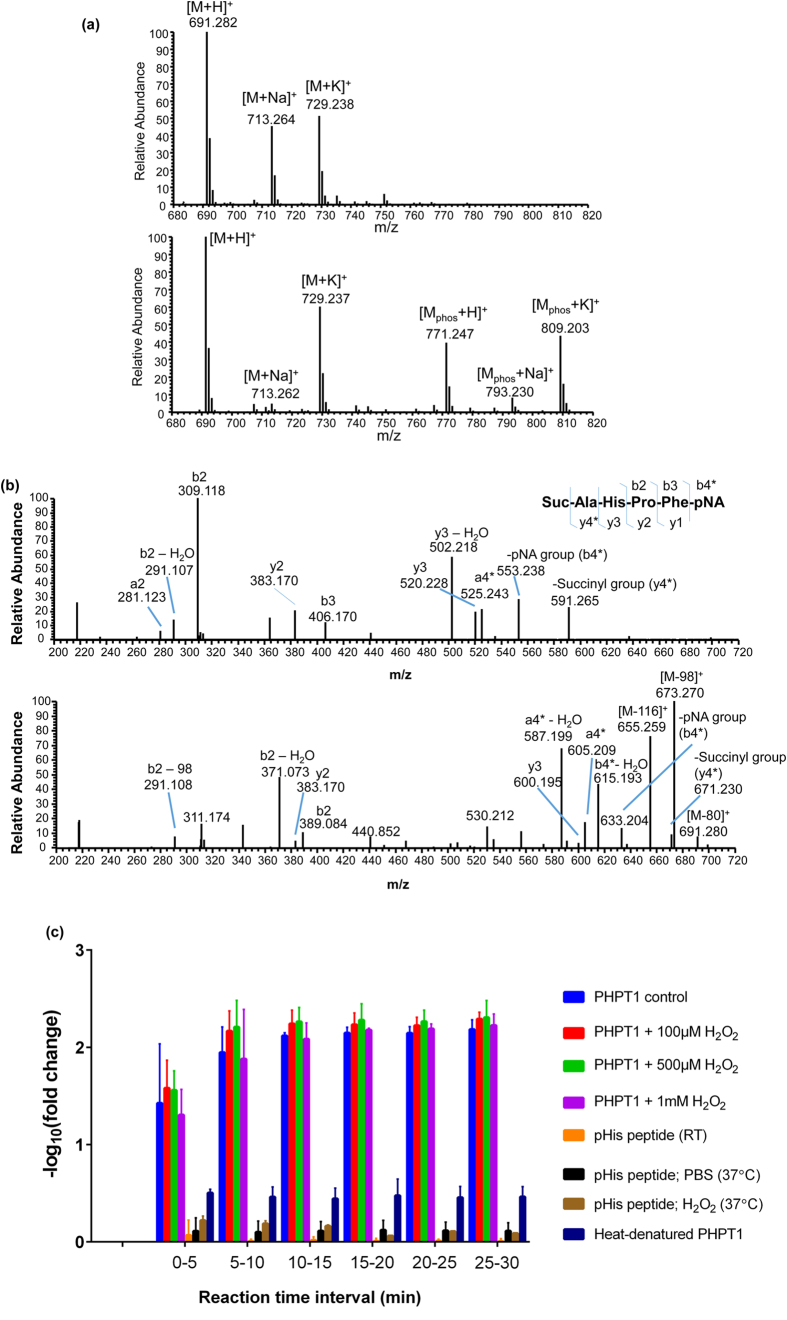
Effect of oxidation on hPHPT1 activity. (**a**) Full scan high-resolution mass spectrum (centroid mode) of the non-phosphorylated peptide (top spectrum), Suc-AHPF-pNA, at *m/z* 691. Signal corresponding to the phosphorylated peptide ion (*m/z* 771) was not detected. Full scan mass spectrum (centroid mode) of the histidine phosphorylated peptide is shown in the bottom spectrum, Suc-A(p)HPF-pNA, at *m/z* 771. Sodium and potassium adducts for both the non-phosphorylated (*m/z* 713 and 729, respectively) and phosphorylated peptide (*m/z* 793 and 809, respectively) are present as well. (**b**) Annotated high-resolution MS/MS spectrum (centroid) of the non-phosphorylated peptide (top spectrum) and phosphorylated peptide (bottom spectrum) confirmed the sequence of the peptide substrate before and after chemical phosphorylation. b- and y-type fragment ions correspond to cleavage of the amide bond at specific locations on the peptide sequence as shown in the inset sequence (*indicates fragment ion corresponding to cleavage of amide bond linking succinyl or pNA to the N-terminus or C-terminus, respectively) (**c**) Mass spectrometry-based activity assay results showing the effect of H_2_O_2_ treatment on phosphohistidine-containing peptide substrate conversion to product by hPHPT1. The −log_10_(fold change) of the phosphorylated peptide was used to demonstrate the amount of the phosphorylated substrate converted to non-phosphorylated product by hPHPT1 relative to the amount of the untreated phosphorylated peptide over time. While the protonated phosphopeptide was not detectable after 10 min, the sodium and potassium adducts of the phosphopeptide allowed for activity measurement at later time points. The activity assay indicated potentially slower conversion of the sodium and potassium adducts. Error bars represent standard deviation from three technical replicates.

The same hPHPT1 samples that were used for oxidation site mapping were assessed for phosphohistidine phosphatase activity using the phosphohistidine containing peptide, Succinyl-Ala-His-Pro-Phe-*p*-nitroanilide, which is a known substrate of PHPT1^9^. The peptide substrate that was not phosphorylated on histidine was used to establish the intrinsic spectral characteristics for this peptide (Fig. 5a, top spectrum). The phosphorylated peptide substrate that was not treated with hPHPT1 was used to determine the maximum amount of phosphorylated histidine present in the samples (Fig. 5a, bottom spectrum). The phosphorylated peptide substrate treated with only 1 mM H_2_O_2_ was used for confirming that the phosphohistidine was not dephosphorylated by H_2_O_2_ addition or that H_2_O_2_ introduced any chemical modifications in the peptide. MS/MS spectra of both the non-phosphorylated and phosphorylated substrate was obtained to confirm sequence and localization of the phosphorylation site (Fig. 5b). In addition to the neutral loss of HPO_3_^−^ (M-80) corresponding to the fragmentation of the labile P-N bond of phosphohistidine, neutral losses of M-98 and M-116 were detected in the MS/MS spectra of the phosphohistidine-containing peptide substrate. The mechanism of gas-phase dissociation of phosphohistidine-containing peptides has been described previously^34^, where the additional water losses arise from fragmentation of the phosphoryl group after gas-phase phosphate transfer from the phosphohistidine to a carboxyl group within the peptide sequence (most likely a phosphate transfer to the N-terminal succinyl group for the phosphopeptide substrate used in this study).

Initial substrate peptide phosphorylation levels were determined to be approximately 35–40% and showed no significant change in phosphorylation when subjected to 1 mM H_2_O_2_ treatment without hPHPT1 or PBS (Fig. 5c). Treatment with non-oxidized hPHPT1 decreased the phosphorylation level of the peptide by 100-fold after 10 min of reaction time (Fig. 5c). No protonated phosphopeptide was detectable after the 10-min time point; however, signal remained for the sodium and potassium adducts, permitting activity measurements at later time points. In addition to the correlation of selective Met95 oxidation that provides evidence of the reported higher order structure of hPHPT1 (Fig. 3), the mass spectrometry-based assay demonstrated robust activity of the recombinant hPHPT1 used in this study. Interestingly, however, hPHPT1 treated with H_2_O_2_ showed no systematic change in phosphatase activity compared to non-oxidized hPHPT1 (Fig. 5c). Heat-denatured hPHPT1 was also used as a negative control and demonstrated a marked decrease, but not total elimination of phosphatase activity, indicating a small amount of hPHPT1 refolds and was active after heat denaturation. The activity assay shows that oxidation of hPHPT1 does not affect its activity, at least with the peptide substrate used in this study.

In addition to the mass spectrometry-based assay, we also used the non-specific phosphatase substrate, para-nitrophenyl phosphate (*p*NPP), to measure product (p-nitrophenolate) generation associated with hPHPT1 activity through colorimetric detection^14^. Both hPHPT1 and 500 μM H_2_O_2_-treated hPHPT1 were used at five different substrate concentrations to determine Michaelis-Menten kinetics of hPHPT1 before and after ROS-induced oxidation. These experiments showed no significant change in the V_max_ and k_cat_ of hPHPT1; however, there was a 1.4-fold decrease in K_m_ following 500 μMH_2_O_2_ treatment (Supplemental Fig. 1).

## Discussion

Identification and quantitation of protein modifications provide insight into protein functional outcomes in response to cellular stress or other stimuli. Additionally, characterizing modifications at site-specific levels establishes causality between structure and function. In this study, using high-performance liquid chromatography combined with high-resolution tandem mass spectrometry, we quantified oxidation of amino acid residues that are susceptible to oxidation (Met, Tyr, Trp, and Cys). The initial unbiased screening using MaxQuant demonstrated greater oxidation levels of the two methionine residues, Met64 and Met95, with significantly higher oxidation of Met95 observed with increased concentration of H_2_O_2_. Upon subsequent analysis using high mass accuracy XIC-based quantitation, we determined that the internally localized methionine residue, Met64, was not significantly oxidized following H_2_O_2_ treatment at concentrations up to 1 mM (maximum 2-fold increase). Met95, however, was oxidized in a concentration-dependent manner, with a site-specific oxidation increase of approximately 40-fold after the 2 hour treatment with 1 mM H_2_O_2_. This result is consistent with the 3D structure of hPHPT1 in that while Met95 is exposed to solvent, Met64 is buried in a partially hydrophobic cavity (Fig. 3d). In general, buried methionine residues are less susceptible to oxidation^35^.

Interestingly, Met95 is also located in the substrate binding site of hPHPT1^16^, indicating a distinct possibility that its oxidation could modulate hPHPT1 activity. In general, oxidative modifications, including those of methionine residues, have been shown to affect protein binding affinity and influence enzymatic activity negatively^36^,^37^. For example, methionine oxidation within calmodulin can negatively affect its ability to activate Ca-ATPase^38^,^39^,^40^. Methionine oxidation has also been shown to affect the function of other proteins such as actin^41^ and IκBα^42^. In the context of PHPT1, a decrease in enzyme activity could prevent removal of phosphoryl groups of its phosphohistidine-containing protein substrates in the cell which include the beta subunit of G protein, ATP-citrate lyase, and the calcium activated potassium channel, KCa3.1. These phosphohistidine targets play vital roles in lipid metabolism and signaling pathways and it is tempting to speculate that alteration of PHPT1 activity, which includes changes due to ROS-induced modifications, could have an impact on these cellular processes.

In principle, Met95 oxidation can influence hPHPT1’s catalytic activity by altering the structure/dynamics of its apo-state or its substrate-bound transition state. We carried out molecular dynamics simulations to predict the effect of Met95 oxidation on the structure/dynamics of hPHPT1’s apo-state. Surprisingly, we found that the apo-state of the catalytic site was affected only marginally by the oxidation state of Met95. Additionally, since NMR studies have revealed no systematic effects of the binding of inorganic phosphate to the catalytic site^16^, we predicted that Met95 oxidation might not affect the phosphate-loaded state of the catalytic site. Therefore, if Met95 oxidation influences hPHPT1 activity, then these studies predict that it would do so by altering the stability of a substrate-bound transition state.

The impact of oxidative modification on hPHPT1 activity was assessed in two different ways. In one approach, we used a mass spectrometry-based activity assay in which a known phosphohistidine-containing peptide substrate of hPHPT1 was utilized. In the second approach, we used a colorimetric assay using the nonspecific substrate, p-nitro phenyl phosphate.

From an experimental standpoint, phosphohistidine has been recognized as an analytical challenge due to the acid labile nature of this modification^5^. We were able to generate a relatively high yield of the known hPHPT1 peptide substrate, Succinyl-Ala-His-Pro-Phe-*p*-nitroanilide (35–40% phosphorylated), that was also stable at 4 °C for months at a time (no significant loss as determined by mass spectrometry). Stability was achieved through solvent composition in which the buffer was at a high pH (pH 8.5–9) and low enough in salt components so as to not interfere with the mass spectrometric analysis. In addition, using a short peptide limited the possibility of alternatively phosphorylated residues and other potential side reactions from the phosphorylation reaction. To the best of our knowledge, this study represents the first time a mass spectrometry-based direct injection technique has been utilized to determine phosphohistidine phosphatase activity. This method is beneficial in that it provides specific detection through molecular weight and sequence information (through MS/MS) of a substrate and product rather than colorimetric or fluorometric assays^43^, which could be nonspecific in detection depending on the matrix composition and complexity. Our reported method could potentially be replicated using other peptide or protein sequences and could also be multiplexed based on the mass resolving power of the mass spectrometer used.

Consistent with predictions from molecular simulations, we found no significant decrease in hPHPT1 activity following oxidation of hPHPT1, as demonstrated by the mass spectrometry-based activity assay. This result is contrary to the common conception that protein oxidation is typically a loss-of-function modification^36^,^37^,^38^,^39^,^40^,^41^,^42^. Furthermore, the colorimetric data demonstrated no significant differences in the k_cat_ or V_max_ of hPHPT1 following H_2_O_2_ treatment; however, the K_m_ value was slightly lower for oxidized hPHPT1. These data could suggest a small increase in substrate binding affinity when the hPHPT1 enzyme is oxidized, although the results are potentially less significant to our assessment of hPHPT1 activity based on the small change in kinetic parameters observed as well as significantly lower specificity of the substrate used in this assay compared to the mass spectrometry-based assay. Based on the results from both assays, we conclude that oxidation of hPHPT1 with concentrations up to 1 mM of H_2_O_2_ did not negatively impact the activity of hPHPT1.

Methionine oxidation has been shown in many cellular roles as a protein modification. Commonly, under conditions of oxidative stress, it is recognized as a ROS-induced modification that typically leads to loss of protein function. Methionine oxidation, however, is not exclusively limited to protein loss of function. For example, it has also been shown that glutamine synthetase in *E. coli* has 8 of its 16 methionine residues oxidized without a significant effect on its enzymatic activity^35^. The role of these multiple oxidation events is believed to be as an antioxidant function (i.e., a ROS “sponge”) in order to protect against the oxidation of redox-sensitive residues of other more sensitive biological targets in ROS-abundant environments. hPHPT1 is unlikely to protect other protein targets in this context given hPHPT1 is a lower abundance protein depending on tissue type^15^,^43^ and only has two methionine sites (one surface exposed) available for modification. It is plausible, though, that selective oxidation of Met95 protects proximal redox-sensitive residues within hPHPT1 that could affect activity, allowing for a functional enzyme even in the presence of high concentrations of ROS.

Although proteome diversity increases significantly through the addition of various post-translational and chemical modifications including oxidative stress-induced modifications such as methionine oxidation, the results from our study provide some evidence that proteome diversity through modifications does not necessarily correlate to functional diversity. Given our studies are limited to a single protein and modification type, it is clear that a significant amount of work is needed to address the functional impact of oxidation as well as other post-translational modifications on a broader scale.

## Conclusion

We have successfully implemented a mass spectrometry-based approach to quantify site-specific oxidation of human PHPT1 (hPHPT1) following treatment with a known cellular ROS, H_2_O_2_. Furthermore, using mass spectrometry, we have shown that selective methionine oxidation at Met95 of hPHPT1 occurred but had no impact on phosphatase activity with the peptide substrate used in this study. Mass spectrometry-based structure and activity experiments were augmented by molecular dynamics simulations that showed methionine oxidation at Met95 had no significant structural impact on the catalytic site of hPHPT1. As our study is limited to small molecule and peptide substrates, other possible outcomes of hPHPT1 (or PHPT1 in other mammalian systems) oxidation need to be explored in a broader context including influence on protein-protein interactions as well as possible changes in cell localization. Future studies aim to characterize PHPT1 structure and expression in various tissue types and conditions of oxidative stress in order to further understand regulation of PHPT1 and related functional consequences in the cell.

## Additional Information

**How to cite this article**: Martin, D. R. *et al*. Structural and activity characterization of human PHPT1 after oxidative modification. *Sci. Rep.* **6**, 23658; doi: 10.1038/srep23658 (2016).

## Supplementary Material

Supplementary Information
