# Supplementary material for: Structural and activity characterization of human PHPT1 after oxidative modification
Source: Sci Rep. 2016 Apr 1;6:23658. doi: 10.1038/srep23658 (PMC4817053; doi:10.1038/srep23658)
Supplement: Supplementary Information [file srep23658-s1.pdf]

Supplemental Information

Structural and activity characterization of human PHPT1 after oxidative modification

Daniel R. Martin, Priyanka Dutta, Shikha Mahajan, Sameer Varma and Stanley M. Stevens Jr.*

Department of Cell Biology, Microbiology and Molecular Biology, University of South Florida, Tampa, FL 33620

*corresponding author; Email: smstevens@usf.edu

Keywords:

Oxidative stress, phosphohistidine, PHPT1, mass spectrometry, molecular dynamics

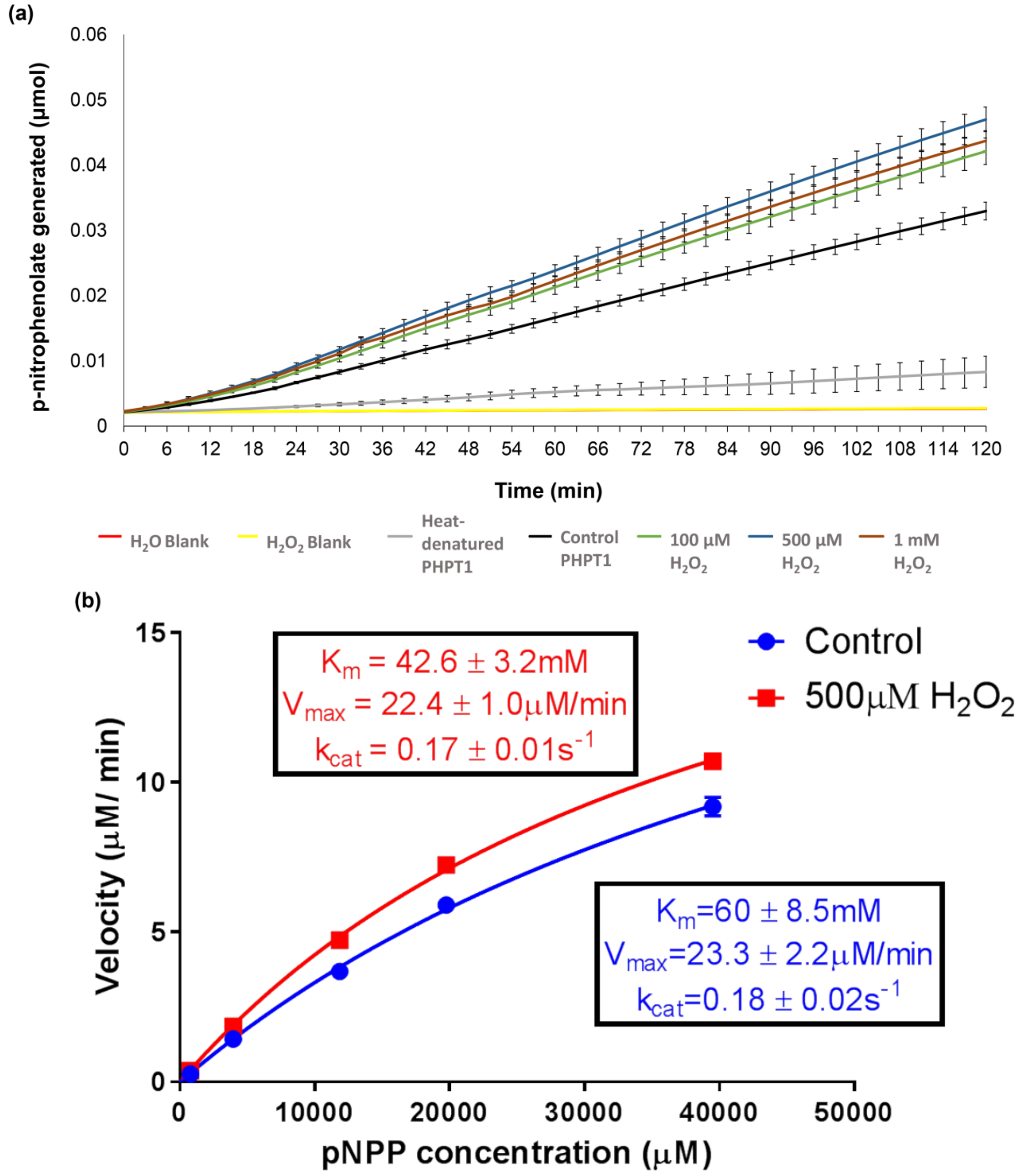

Supplemental Figure 1. Colorimetric phosphatase activity assay results using the p-nitrophenyl phosphate (*p*NPP) substrate for PHPT1 before and after H_2O_2 treatment. **(a)** Representative colorimetric assay reading over 2 h at 5 mM initial pNPP concentration where error bars represent standard deviation. Error bars represent standard deviation of plated and technical replicates (n=9). **(b)** Nonlinear regression using the Michaelis-Menten equation was performed for control and 500 µM H_2O_2-treated PHPT1 with a substrate concentration range from 0.8mM-40mM. K_m, V_{max}, and k_{cat} values are shown with error representing 95% confidence intervals.
